# MetaboVariation: Exploring Individual Variation in Metabolite Levels

**DOI:** 10.3390/metabo13020164

**Published:** 2023-01-23

**Authors:** Shubbham Gupta, Isobel Claire Gormley, Lorraine Brennan

**Affiliations:** 1 School of Agriculture and Food Science, University College Dublin, Belfield, D04 V1W8 Dublin, Ireland; 2 School of Mathematics and Statistics, University College Dublin, Belfield, D04 V1W8 Dublin, Ireland

**Keywords:** metabolite levels, intra-individual variation, Bayesian generalised linear model, personalised healthcare

## Abstract

To date, most metabolomics biomarker research has focused on identifying disease biomarkers. However, there is a need for biomarkers of early metabolic dysfunction to identify individuals who would benefit from lifestyle interventions. Concomitantly, there is a need to develop strategies to analyse metabolomics data at an individual level. We propose “MetaboVariation”, a method that models repeated measurements on individuals to explore fluctuations in metabolite levels at an individual level. MetaboVariation employs a Bayesian generalised linear model to flag individuals with intra-individual variations in their metabolite levels across multiple measurements. MetaboVariation models repeated metabolite levels as a function of explanatory variables while accounting for intra-individual variation. The posterior predictive distribution of metabolite levels at the individual level is available, and is used to flag individuals with observed metabolite levels outside the 95% highest posterior density prediction interval at a given time point. MetaboVariation was applied to a dataset containing metabolite levels for 20 metabolites, measured once every four months, in 164 individuals. A total of 28% of individuals with intra-individual variations in three or more metabolites were flagged. An R package for MetaboVariation was developed with an embedded R Shiny web application. To summarize, MetaboVariation has made considerable progress in developing strategies for analysing metabolomics data at the individual level, thus paving the way toward personalised healthcare.

## 1. Introduction

Nutrition research has made significant progress in recent years. Though it is widely accepted that early lifestyle interventions could reduce disease burden, it is difficult to identify individuals that would benefit most from targeted nutrition interventions. While metabolomic approaches have been highly successful in identifying disease biomarkers [1,2,3], there is a lack of biomarkers related to early metabolic disturbances. Successful areas to date include the study of acylcarnitines in various diseases—there is strong evidence showing alterations in acylcarnitine levels in metabolic disorders, cardiovascular diseases, diabetes, depression and neurologic disorders [4]. In the last 10 years, accumulating evidence has supported the role of branched-chain aminos acids as biomarkers for the development of type 2 diabetes. Recently, a systematic review and meta-analysis reported that plasma branched-chain amino acids were associated with type 2 diabetes incidence, irrespective of the time of follow-up [5]. Though branched-chain amino acids present an interesting proof of concept for biomarkers of early metabolic dysfunction, it is essential to explore alternative approaches. Concomitant with this is the need to embrace personalised approaches and analyse metabolomics data at the individual level [6]. This is driven partly by the emergence of personalised nutrition approaches, where dietary advice is tailored to the individual [7].

Recent research [8] has demonstrated that individual variation in blood metabolites may help discover potential indicators of human ageing or pertinent diseases. Metabolites represent individual variations in human biological samples. Using metabolomics data from whole blood, a total of 48 metabolites showed moderate to high coefficients of variations (CVs); the authors [8,9,10] proposed these metabolites as potential biomarkers and put forward the concept that metabolites with high CVs could be considered personal markers. However, many factors such as age, BMI, nutrition, ethnicity, gender or lifestyle impact the metabolome, and there is a need to develop methods to examine metabolites at an individual level. Furthermore, considering these and other factors, such as diet, it is challenging to establish workable reference ranges for the metabolome. Hence, the concept of monitoring an individual and the variation within an individual has emerged. Using repeated measures from individuals can highlight deviations from one’s typical profile and can indicate the onset of metabolic perturbations [11]. Applying this concept using omics technologies over a 14-month monitoring period [12] demonstrated the presence of dynamic alterations in profiles as a participant moved from a healthy to diseased state. In a follow-up study, the longitudinal signature of omics data was related to insulin resistance, with the data indicating that each individual was unique, calling for the need to analyse data at the level of the person [13]. However, there is a paucity of statistical approaches that identify individuals with small metabolic perturbations and that model metabolomic data at the individual level.

The objective of the present study was to develop a method called MetaboVariation that uses a Bayesian generalised linear model (BGLM) to flag individuals with intra-individual variations in their metabolite levels. To accomplish this, we modeled repeated measures of individuals’ metabolite levels to infer typical fluctuations of metabolites in individuals. Given our objective, the Bayesian paradigm is particularly advantageous due to its natural provision of posterior predictive distributions; after inferring typical fluctuations, posterior predictive distributions are used to flag individuals with intra-individual variability in their metabolite levels. The novelty of the MetaboVariation approach is twofold: firstly, it models metabolomics data at an individual level and secondly, it flags individuals with intra-individual variation in their metabolite levels.

The statistical details underpinning MetaboVariation are described in Section 2. Section 3 assesses the performance of MetaboVariation and discusses the results using simulated and real-world data. Section 4 discusses the use of MetaboVariation and potential enhancements, and our conclusions are in Section 5. All reported results were created using the R package MetaboVariation (https://github.com/shubbham28/MetaboVariation, accessed on 20 December 2022), which is freely accessible. An R Shiny web application is available within the R package to aid user accessibility.

## 2. Materials and Methods

In metabolomics, repeated measurements of individuals are frequent, but their analysis can be complex. Despite the availability of a range of specialised analytic tools, such as generalised linear models (GLMs) [14], there has not been widespread use of such tools to model repeated measures metabolomics data.

### 2.1. The MetaboVariation Method

In this study, we propose MetaboVariation, which employs a Bayesian generalised linear model (BGLM) to model repeated measures of metabolite levels of individuals. MetaboVariation is fitted using the MCMCglmm package [15], which uses Metropolis–Hastings updates [16]. In the following, we assume independence between the individuals in the cohort whose metabolite levels are being measured, and between the metabolites. The model considers metabolomics data that contain the levels of *M* metabolites for *N* individuals across *T* time points. We consider a single metabolite *m*, recorded in an N×T matrix $Y^{m}$. The covariates of individuals are stored in an N×L matrix X, where *L* denotes the number of covariates. The level of metabolite *m* of a single individual *i* at time point *t* is modelled as

(1) $$y_{it}^{m}=\beta_{0}+\sum_{l=1}^{L}\beta_{1,l}x_{il}+s_{it}+\epsilon_{it}$$

where $y_{it}^{m}$ is the level of metabolite *m* for individual *i* at time point *t* and $x_{il}$ is covariate *l* for individual *i*. The mean intercept is denoted by $\beta_{0}$, whereas $\beta_{1,l}$ is the regression coefficient for covariate *l*. The random effect for the $i^{th}$ individual at time point *t* is denoted by $s_{it}$, where $s_{it}∼N\left(0,\sigma^{2}\right)$, and $\epsilon_{it}$ is the random error for individual *i* at time point *t* where $\epsilon_{it}∼N\left(0,\sigma_{\epsilon }^{2}\right)$. Here, *S* and ϵ respectively denote N×T matrices of random effects and random errors for *N* individuals at *T* time points. However, as metabolite levels among individuals will differ, the intercept should also differ for different individuals, i.e., $\beta_{0,i}\neq \beta_{0,j}$ for individuals *i* and *j*. To simplify the model, for every individual *i*, the difference $\beta_{0,i}-\beta_{0}$ is incorporated into the term $s_{it}$, thus not influencing the estimates of $\beta_{0}$ and $\beta_{1}$ where $\beta_{1}=\left(\beta_{1,1},\beta_{1,2},...,\beta_{1,L}\right)$. As (1) considers only a single metabolite *m*, correlation among metabolites is not considered here.

The posterior distribution of the BGLM, given the covariates X and metabolite data $Y^{m}$, is:

(2) $$P\left(\beta_{0},\beta_{1},\sigma^{2},\sigma_{\epsilon }^{2}|Y^{m},X\right)\propto P\left(Y^{m}|\beta_{0},\beta_{1},\sigma^{2},\sigma_{\epsilon }^{2},X\right)\times P\left(\beta_{0},\beta_{1},\sigma^{2},\sigma_{\epsilon }^{2}\right)$$

where the first term on the right-hand side of (2) is the likelihood function of the data and the second term denotes the assumed prior distributions of the regression parameters $\beta_{0}$ and $\beta_{1}$ and variance parameters $\sigma^{2}$ and $\sigma_{\epsilon }^{2}$. Given the model, the likelihood is Gaussian. Independent prior distributions are assumed for the regression and variance parameters: zero-centred Gaussian priors with large variance (1e + 10) are assumed for the regression parameters with inverse Wishart prior distributions assumed for the variance parameters, following the default settings in the MCMCglmm package [15].

Samples of $\beta_{0},\beta_{1},\sigma^{2}$ and $\sigma_{\epsilon }^{2}$ are drawn from the posterior distribution using a Metropolis–Hastings algorithm and are used to predict individuals’ metabolite levels. A posterior predictive distribution is the distribution of new data points conditional on the observed data [17]. To construct the posterior predictive distribution for an individual *i* at time point *t*, several linear models are constructed by drawing model parameters and latent variables from their joint posterior distribution. The number of models is equal to the number of chains multiplied by the difference between the number of iterations and burn-in iterations. Each model gives a predicted value, thus creating samples from the posterior predictive distribution of ${\tilde{y}}_{it}^{m}$, where ${\tilde{y}}_{it}^{m}$ denotes the predicted level of metabolite *m* for individual *i* at time point *t*.

After drawing samples from the posterior predictive distribution $p({\tilde{y}}_{it}^{m})$, a W% highest posterior density (HPD) prediction interval is formed, where typically W=95. If the observed value $y_{it}^{m}$ lies outside the HPD prediction interval, the individual *i* at time point *t* is flagged, suggesting that the individual *i* has intra-individual variability in their level of metabolite *m* at time point *t* compared with their metabolite levels at other time points.

In order for the posterior predictive distributions to be valid, the Metropolis–Hastings algorithm must have converged such that independent samples of $\beta_{0},\beta_{1},\sigma^{2}$ and $\sigma_{\epsilon }^{2}$ have been drawn from the posterior distribution. Assessing the convergence of the Metropolis–Hastings algorithm and the independence of the samples drawn is therefore necessary. Though a range of diagnostic tools are available to assess convergence [17], for this study, we employed the potential scale reduction factor (PSRF). The PSRF relies on running multiple Metropolis–Hastings algorithms or chains for each model fitted, where the starting points for each chain are dispersed throughout the parameter space. Here, the starting values for the multiple chains follow the default settings in the MCMCglmm package [15]. The PSRF approach considers the estimated between- and within-chain variances for each model parameter, with approximate equality indicating possible convergence. For each model parameter, PSRF values in the range 0.9 to 1.05 are typically used to indicate convergence. Here, PSRF values are monitored for the $\beta_{0},\beta_{1},\sigma^{2}$ and $\sigma_{\epsilon }^{2}$ parameters of the model. To ensure independence of the sampled model parameters, informed by autocorrelation plots, the chains were thinned every second iteration.

To prevent overfitting, the total number of observations for each metabolite (i.e., N×T metabolite levels) was divided into twenty-five sub-datasets (i.e., 4% of observations in each sub-dataset). A BGLM was fitted to all observations with one sub-dataset held out and the posterior predictive distributions were constructed for the held-out sub-dataset. The process of fitting the BGLM while holding out one sub-dataset was repeated for all sub-datasets, thus constructing posterior predictive distributions for all N×T metabolite levels.

The data used in the study was taken from the A-Diet Confirm study [18,19], for which ethical approval was granted by the University College Dublin Sciences Human Research Ethics Committee (LS-16-91-Gibbons-Brennan). Briefly, the A-Diet Confirm study was designed to examine the habitual dietary intake of participants during a four month period with collection of biological samples and dietary data once a month. For the present study, the amino acid data was used from the previously reported dataset [18]. The data was acquired using the Biocrates AbsoluteIDQ p180 kit on a Sciex QTRAP 6500+ mass spectrometer coupled to a UHPLC column. The amino acids were quantified using isotopically labelled internal standards and seven-point calibration curves in AB Sciex Analyst version 1.7.2 software. In the quality control analysis, all amino acids had CV% less than 20% in pooled samples [18].

### 2.2. Simulation Study Design

Different simulation scenarios were constructed to explore the performance of MetaboVariation. Every scenario considered different settings of the parameters α and ν, where a proportion α of individuals was selected, and for each selected individual, the variation in one of their randomly selected repeated time points was inflated by the factor ν. For each scenario, 30 datasets were simulated to assess robustness of the method.

The simulated data were constructed such that they were similar to the real-life data of fasting plasma samples from the A-Diet Confirm study [18,19]. Each simulated dataset contained four repeated measurements of a single metabolite for 150 individuals with three covariates. Two covariates were simulated from Gaussian distributions, with means and standard deviations estimated from the age and BMI covariates in the real data of fasting plasma samples; similarly, the third covariate was simulated from a binomial distribution with a similar male to female ratio as in the real data of fasting plasma samples. A BGLM was fitted to alanine metabolite levels from the real data using the three simulated covariates, age, sex and BMI. The estimates of the regression coefficients ${\hat{\beta }}_{0}$ and ${\hat{\beta }}_{1}$, error variance (${\hat{\sigma }}_{\epsilon }^{2}$) and variance at an individual level (${\hat{\sigma }}^{2}$) were used along with the simulated covariates to simulate metabolite levels across four repeated measurements using (1), where $s_{it}∼N\left(0,{\hat{\sigma }}^{2}\right)$ and $\epsilon_{it}∼N\left(0,{\hat{\sigma }}_{\epsilon }^{2}\right)$.

Once the metabolite levels were simulated, a metabolite level was generated from $N(0,{\hat{\sigma }}^{2}\nu )$, where ν was the inflation factor. This metabolite level was added to the existing metabolite level at one randomly selected time point for each of the randomly selected proportion α of individuals. We ensured that the inflated metabolite levels lay outside the [1,99%] interval of the non-inflated metabolite levels. After forming the simulated dataset, MetaboVariation was fitted to the data and the number of flagged individuals with inflated variance was assessed. This process was repeated over the 30 simulated datasets.

## 3. Results

**Figure 1:**
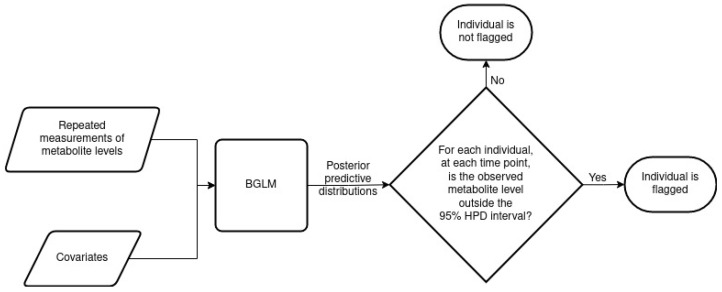
An overview of the MetaboVariation method.

MetaboVariation analyses repeated metabolite measurements and flags individuals with different levels in at least one of these repeated measurements. Before detailing our results, we provide an overview of the MetaboVariation method. Using repeated measures from individuals, MetaboVariation uses a Bayesian generalised linear model (BGLM) that models metabolite levels as a function of covariates while accounting for intra-individual variation. If no covariates are provided, the model will only consider the repeated measurements of metabolite levels. The BGLM generates a posterior predictive distribution for each individual at each time point. When an individual’s observed metabolite level falls outside a specified percentage, e.g., the 95% highest posterior density (HPD) prediction interval, at a given time point, the model flags that particular individual as having intra-individual variation in their metabolite levels. Figure 1 provides an overview of the MetaboVariation method.

### 3.1. Simulation Study

The objective of the simulation study was to assess the robustness and performance of the MetaboVariation method. Thirty simulated datasets were created, each having four repeated measures of one metabolite from 150 individuals with three covariates (see Section 2.2 for specific details). A proportion α of individuals was selected, and for each selected individual, the variation in one of their randomly selected repeated measures was inflated by the factor ν. Varying the values of α and ν allows assessment of the robustness and performance of MetaboVariation; α∈{10%,20%} and ν∈{5,10,25} were considered here. The performance of MetaboVariation was assessed through examination of the proportion of individuals with known intra-individual variations that were correctly flagged across the 30 simulated datasets.

At α=10%, when ν was increased from 5 to 10 to 25, the mean proportion (standard deviation in parentheses) of individuals flagged correctly was 0.97 (0.05), 0.96 (0.04) and 0.95 (0.05), respectively. These results suggest that across different variance inflation factors, when 10% of the individuals have intra-individual variation in their metabolite levels, MetaboVariation is able to correctly flag individuals that have intra-individual variability in their metabolite levels.

At α=20%, the mean proportion (standard deviation in parentheses) of individuals flagged correctly was 0.9 (0.06), 0.89 (0.05) and 0.87 (0.05) for a ν of 5, 10 and 25, respectively. These results imply that as the proportion of individuals that have intra-individual variation in their metabolite levels increases, it is more difficult for MetaboVariation to discriminate between increased and minor intra-individual variation.

For every setting of α and ν for each of the 30 simulated datasets, convergence was assessed by running four chains and calculating the PSRF for each of the four parameters, $\beta_{0},\beta_{1},\sigma^{2}$ and $\sigma_{\epsilon }^{2}$. Across all cases, the average PSRF was 1.0003 (standard deviation 0.0004).

### 3.2. Case Study: Fasting Plasma Samples from A-Diet Confirm Study

Fasting plasma samples from the A-Diet Confirm study [18,19] were collected monthly over four consecutive months in a free-living population. For this study, twenty metabolites were used from the original data and all 164 participants had at least two repeated measures. The participants had an average BMI of 24.1±3.07 kg/m${}^{2}$ and average age of 35±12.6 years. Data from a total of 53 men and 111 women were analysed.

**Figure 2:**
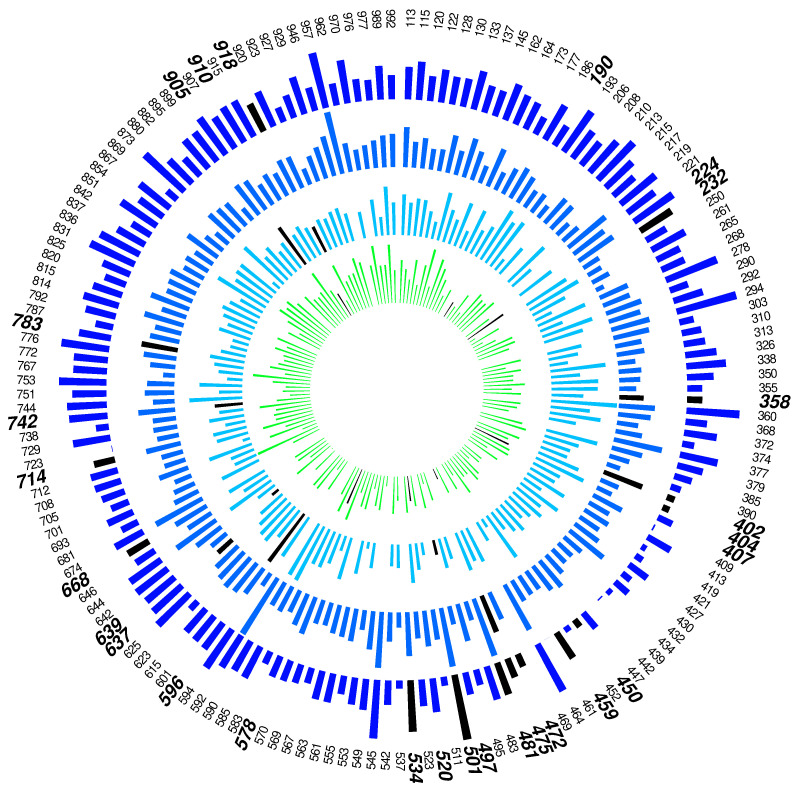
Circos plot for alanine. Each individual’s label is shown in the outer circle, with bold font indicating those who have been flagged. The length of a bar within a time point has been scaled to represent the width of the central 95% posterior predictive interval. Flagged individuals have a black bar at the flagged time point. No bar indicates that the individual had no data for that time point.

When MetaboVariation was applied to the data, 28% of individuals were flagged as having intra-individual variation in at least three of the twenty metabolites. A circos plot for the metabolite alanine is shown in Figure 2.

There was a significant relationship between several metabolites and sex, whereas some metabolites had a significant relationship with age and BMI. Table 1 shows the estimates of the regression coefficients for sex, age and BMI for each metabolite, with 95% credible intervals. To assess convergence, four chains were run for each of the twenty metabolites, with an average PSRF of 1.0003 (standard deviation 0.0003) for each of the parameters ($\beta_{0}$, $\beta_{1}$, $\sigma^{2}$ and $\sigma_{\epsilon }^{2}$).

**Figure 3:**
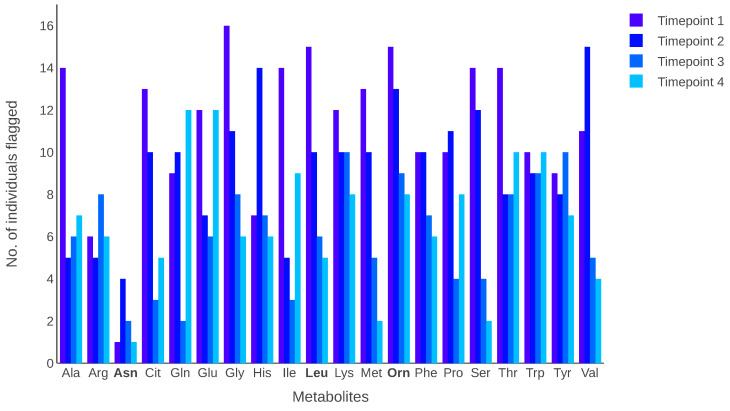
The number of individuals flagged for each metabolite. For each metabolite, a bar indicates a different time point. Bold font indicates metabolites with the highest or lowest number of individuals flagged across the four time points.

Figure 3 depicts the number of individuals flagged in each metabolite at each time point. The results indicate that some metabolites had many individuals with intra-individual variation in their metabolite levels. When individuals are flagged, they are counted only once, even if they are flagged at more than one time point for a metabolite. The metabolites leucine and ornithine had the highest number of individuals flagged across the four time points, whereas the metabolite asparagine had the lowest number of flagged individuals.

MetaboVariation was used on a Dell Latitude 5511 machine, running on Ubuntu 20.04 with an Intel 10th Gen i7 core and 16 GB memory. Applying MetaboVariation on a single metabolite, running four different chains with 5000 iterations each and 2500 burn-in iterations, took approximately 2 minutes. Execution time varies depending on the system, number of chains, number of iterations and the number of individuals present in the data.

## 4. Discussion

There has been an increased interest in personalised healthcare, where an individual receives dietary advice tailored to their characteristics [20,21,22,23,24]. Omics technologies, such as metabolomics, metagenomics, proteomics and transcriptomics, have recently become highly relevant in pursuing these goals [25]. For metabolomics data, we developed MetaboVariation to flag individuals with intra-individual variations in their metabolite levels. To address this problem, we present a conceptual method that employs repeated measurements of individual metabolite levels to understand intra-individual variances, and flag those with significant variations in their metabolite levels. An advantage of MetaboVariation is that it does not rely on generalised metabolite ranges and models the data at an individual level while also considering covariates. Further, an R package “MetaboVariation” was developed in conjunction with an R shiny application to facilitate widespread use of the method.

With a focus on personalised medicine, a team of researchers [26] developed the individual reference interval (IRI), which provides an interval for a particular variable based on an individual’s biological characteristics. Population reference intervals (PRI) [27], which are derived from a healthy reference population, are crucial for interpreting clinical laboratory tests. Clinical choices today are typically binary: if the observation falls within the PRI, it is deemed normal. The IRI range adds a personal context, extending this interpretation to the individual level. Individual reference intervals describe test findings that, in the case of a healthy individual, would be anticipated to have a probability of about 95%. The key benefit of our study is the absence of a general range underpinning the prediction of whether or not an individual had intra-individual variations in their metabolite levels. This work has potential for the development of personalised healthcare. Continuous monitoring is becoming an embedded aspect of personalised healthcare, with the development of sensors such as continuous glucose monitors. With respect to metabolomics data, repeated measures from individuals over time offer an opportunity to identify the onset of metabolic perturbations and signal for the need of lifestyle interventions. The ability to do this at an individual level, without the need for a population reference value, indicates an important step forward for the development of personalised approaches.

Using the plasma data from 164 individuals [18], the two metabolites leucine and ornithine had the highest number of individuals flagged by MetaboVariation, whereas the metabolite asparagine had the lowest number of individuals flagged. Previous work [28] reported an excellent ICC for asparagine across two measurements. This agrees well with the low frequency of selection of individuals for asparagine by the MetaboVariation method. With respect to the two metabolites with the highest number of individuals flagged, the ICCs previously reported were good, indicating that our method is capable of picking up individual variation that may be missed with an ICC approach [28,29].

Though the present work represents a significant step forward, some limitations do exist. For example, MetaboVariation fits a single metabolite at a time, thus neglecting the correlations between different metabolites. Future work should aim to incorporate these correlations into the model. Further, MetaboVariation assumes that metabolite levels are Gaussian-distributed at each time point, and this assumption may be invalid in some cases. Future work could consider more flexible versions of the MetaboVariation method where a variety of distributions for metabolite levels are permitted. Though the computational costs of fitting the MetaboVariation method to a single metabolite is of the order of minutes, fitting it to larger datasets with high numbers of metabolites could become computationally prohibitive; approximate Bayesian inference approaches could assist in easing this burden.

## 5. Conclusions

In this study, we developed MetaboVariation, a method that uses the repeated measures of metabolite levels in individuals to understand intra-individual variations and flag individuals with significant variations in their metabolite levels. We examined the performance of MetaboVariation through a simulation study and, on applying MetaboVariation to real data, individuals with intra-individual variations in their metabolite levels were flagged. These findings make substantial progress in developing strategies for analysing metabolomics data at an individual level, thus paving the way toward personalised healthcare. Implementation of MetaboVariation by the wider community is made possible through the provision of open-source software.

## Figures and Tables

**Table 1:** Estimates of all regression coefficients for each metabolite, with 95% credible intervals in parentheses.

Metabolite	Sex	Age	BMI
Ala	−4.41 (−26.17, 17.41)	−0.57 (−1.35, 0.2)	2.65 (−0.69, 5.95)
Arg	**−5.49** (−10.35, −0.65)	**0.19** (0.01, 0.37)	−0.64 (−1.41, 0.14)
Asn	−1.32 (−4.8, 2.17)	**−0.16** (−0.28, −0.03)	−0.27 (−0.82, 0.29)
Cit	**−3.82** (−5.81, −1.77)	**0.13** (0.05, 0.2)	−0.3 (−0.61, 0.02)
Gln	**−38.53** (−61.06, −16.57)	**1.57** (0.79, 2.36)	−3.03 (−6.46, 0.46)
Glu	**−8.38** (−13.07, −3.73)	**0.26** (0.1, 0.43)	**1.35** (0.63, 2.08)
Gly	15.45 (−9.12, 40.1)	**0.95** (0.06, 1.85)	−2.61 (−6.51, 1.34)
His	-0.89 (−4.26, 2.56)	−0.06 (−0.18, 0.06)	0.37 (−0.17, 0.89)
Ile	**−19.65** (−23.48, −15.77)	**−0.26** (−0.4, −0.12)	**0.79** (0.19, 1.37)
Leu	**−40.57** (−47.84, −33.33)	**−0.27** (−0.53, −0.02)	**1.71** (0.57, 2.87)
Lys	-8.88 (−17.9, 0.12)	**0.36** (0.03, 0.69)	−0.54 (−1.92, 0.86)
Met	**−2.92** (−4.14, −1.7)	−0.04 (−0.09, 0)	0.01 (−0.18, 0.2)
Orn	**−9.88** (−13.94, −5.88)	**0.29** (0.15, 0.44)	**−0.83** (−1.45, −0.21)
Phe	**−6.19** (−9.23, −3.1)	**−0.11** (−0.22, 0)	0.35 (−0.13, 0.81)
Pro	**−37.61** (−53.97, −21.57)	−0.37 (−0.95, 0.2)	2.37 (−0.22, 4.91)
Ser	**7.51** (0.17, 14.86)	−0.15 (−0.41, 0.11)	−0.87 (−1.99, 0.25)
Thr	4.06 (−3.8, 11.91)	**−0.34** (−0.62, −0.06)	−0.38 (−1.6, 0.8)
Trp	**−8.06** (−11.12, −4.95)	**−0.17** (−0.29, −0.06)	0.33 (−0.17, 0.81)
Tyr	**−5.33** (−9.31, −1.4)	0.07 (−0.08, 0.21)	**0.95** (0.33, 1.57)
Val	**−34.47** (−43.8, −25.42)	−0.06 (−0.41, 0.27)	1.21 (−0.25, 2.64)

Bold font indicates regression coefficients whose 95% credible interval does not include zero.

## Data Availability

Restrictions apply to the availability of these data. They were obtained from the A-Diet Confirm study [18,19] and can be accessible through request from the corresponding author L.B.
